# Effects of Helix Geometry on Magnetic Guiding of Helical Polymer Composites on a Gastric Cancer Model: A Feasibility Study

**DOI:** 10.3390/ma13041014

**Published:** 2020-02-24

**Authors:** Yongju Kim, Jeong Eun Park, Jeong Jae Wie, Su Geun Yang, Don Haeng Lee, Young-Joo Jin

**Affiliations:** 1 Department of Polymer Science and Engineering, Inha University, Incheon 22212, Korea; 22191115@inha.edu (Y.K.); 22171192@inha.edu (J.E.P.); 2 Department of New Drug Development, Inha University, School of Medicine, Incheon 22212, Korea; sugeun.yang@inha.ac.kr; 3 Division of Gastroenterology, Department of Internal Medicine, Inha University Hospital, Inha University School of Medicine, Incheon 22332, Korea; ldh@inha.ac.kr; 4 The National Center of Efficacy Evaluation for the Development of Health Products Targeting Digestive Disorders (NCEED), Incheon 22332, Korea; 5 Utah-Inha DDS & Advanced Therapeutics Research Center, Incheon 22332, Korea

**Keywords:** helical soft robots, magnetic flux density, magnetic composites, gastric cancer, geometric parameters

## Abstract

This study investigates the effects of soft-robot geometry on magnetic guiding to develop an efficient helical mediator on a three-dimensional (3D) gastric cancer model. Four different magnetically active helical soft robots are synthesized by the inclusion of 5-μm iron particles in polydimethylsiloxane matrices. The soft robots are named based on the diameter and length (D2-L15, D5-L20, D5-L25, and D5-L35) with samples having varied helical pitch and weight values. Then, the four samples are tested on a flat surface as well as a stomach model with various 3D wrinkles. We analyze the underlying physics of intermittent magnetomotility for the helix on a flat surface. In addition, we extract representative failure cases of magnetomotility on the stomach model. The D5-L25 sample was the most suitable among the four samples for a helical soft robot that can be moved to a target lesion by the magnetic-flux density of the stomach model. The effects of diameter, length, pitch, and weight of a helical soft robot on magnetomotility are discussed in order for the robot to reach the target lesion successfully via magnetomotility.

## 1. Introduction

Approximately 1,000,000 gastric cancers were newly diagnosed worldwide in 2012 [1], and the incidence of gastric cancer is the highest in Eastern Asia, Eastern Europe, and South America [2,3]. In Korea and Japan, where the incidence of gastric cancer is much higher than that in Western countries [2,3,4], routine gastric cancer screening is conducted via upper gastrointestinal endoscopy [5,6]. However, patients are often struggling with repeated gastroscopic examinations despite the improvements in endoscopic probes in terms of higher flexibility and smaller diameter for patient convenience [7]. This has led to a need for the development of diagnostic or therapeutic methods for gastric cancer using very small-size equipment for patient convenience.

Recently, there has been an increased interest in the development of micro equipment to identify target lesions in narrow spaces inside the body that have previously not been accessible with the currently used medical device technology [8,9,10]. The ability to artificially determine the location of the small substance to be delivered to the target lesion using an externally applied force can significantly assist in the diagnosis and therapeutic treatment of gastric cancer. In an effort to replace the tethered flexible endoscopes, untethered pill-sized capsule endoscopes with a wireless video-transmission device have been used [11,12]. This device enables access to areas that were previously inaccessible and reduces the discomfort caused to the patient such as in the cases of odynophagia and aspiration as well as risks linked to sedation during the examination. However, the capsule endoscopes have some major drawbacks in that the clinician cannot control the position, orientation, or function of the capsule. Thus, the acquisition of an image is inevitably passive, and the chance remains that the images captured will be insufficient in number and quality. Researchers have recently developed active robotic capsules that can be controlled remotely and can actively capture images; [13,14,15] however, these studies are limited to hard and rigid robotic capsules. Given that hard robots offer limited movements over given obstacles in their direction of movement, there is a need for the development of soft robots that can maneuver easily through physical obstacles. Therefore, composite materials containing elastic polymer and magnetic materials were employed with the advantage of having nondestructive property inherent in magnetic field stimulus [16,17,18,19]. Furthermore, helical robots, prepared by dip-coating originally helical bacteria [20] or dynamic light writing process [21,22], have been recently demonstrated to achieve targeted drug delivery. The motility of robots was mainly consigned to swimming, inspired by bacterial flagella. Helical morphology aroused linear translational motion converted from the rotational motions of the helix such as the corkscrew motion. In a liquid environment with a low Reynolds number, it could perform effective propulsion via reduced resistance. However, motility in a dry environment is also required for getting out of limited circumstances within the living body. In the case of the stomach, a number of gastric folds, acting as obstacles, have to be overcome in order to deliver therapeutic substances to the targeted injury in the gastrointestinal environment. We suggest the rolling motility of helical soft robots, whose helixes reduce rolling resistance when compared to cylindrical geometry due to its lower mass [23,24,25]. Two-step polymerization using non-toxic and biocompatible polydimethylsiloxane (PDMS) elastomer is a strength for the facile preparation of helix angle and pitch [26]. More specifically, significant improvements in the diagnosis and treatment of gastric cancer can be achieved if this technique can cost-effectively diagnose early gastric cancer in high-risk populations. However, the challenge has been in determining the efficiency with which a helical object can be delivered to a target lesion, such as a cancer or an ulcer, inside the stomach. 

The aim of this experimental feasibility study is to develop a helical mediator that efficiently delivers a specific object at a target lesion using magnetic force in a gastric cancer model. In addition, an attempt is made to determine the optimal conditions for its shape such that it has the ability to overcome the obstacles within the corrugated stomach by assessing its motion.

## 2. Materials and Methods

### 2.1. Materials

**Figure 1:**
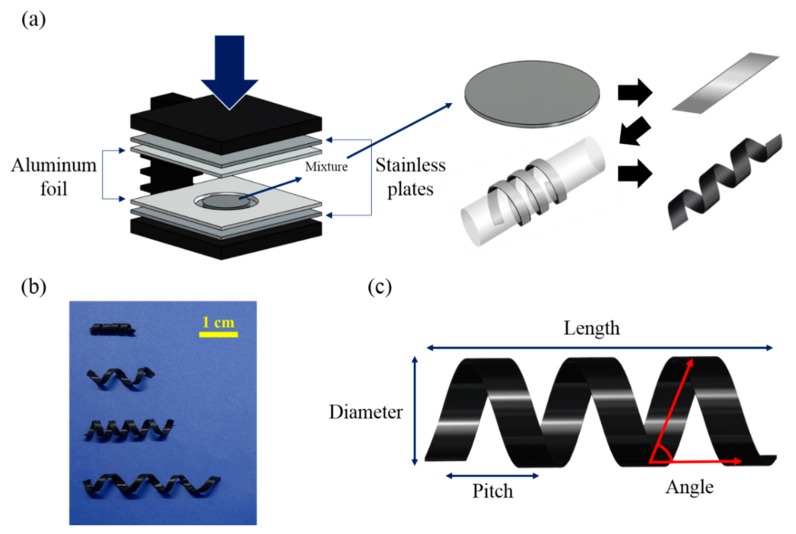
(**a**) Schematic fabrication process for magnetic helical soft robots comprising 3 vol% CIP (carbonyl iron particle) embedded in PDMS (polydimethylsiloxane) matrices (**b**) Digital images of four different samples (D2-L15, D5-L20, D5-L25, and D5-L35 from top to bottom) (**c**) Scheme for illustrating parameters of the helix geometry. Note: The names of the robot samples correspond to the diameter (D) and length (L) (in mm) of their helical coils.

As shown in Figure 1a, magnetically active helical soft robots were synthesized by the two-step polymerization of polydimethylsiloxane (PDMS, Sylgard 184, Dow corning, Midland, MI, USA) 5-µm iron-particle (CIP CC, BASF, Ludwigshafen, Germany) composites. First, a mixture of 3 vol% iron particles and PDMS pre-polymer was degassed at 600 psi for 10 s and precured at 80 °C for 5 min with a 0.4-mm aluminum foil spacer. Then, the precured polymer composite film was cut into 1.5-mm-wide strips. The strips were wrapped around a cylinder with a controlled helical pitch. Finally, the helical coil was fully cured at 80 °C for 1 h and harvested from the cylinder [26]. 

Four different samples with varying geometric parameters were synthesized, including the diameter, length, pitch, and weight of the helical samples (Table 1, and Figure 1b,c). In Table 1, the distance between the threads is named pitch. Moreover, the vertical height of the sample is defined by diameter and the horizontal end-to-end distance is expressed in length. The code names for the four different soft robots were assigned as D2-L15, D5-L20, D5-L25, and D5-L35 (Table 1) based on the diameter (D) and length (L) (in mm) of each helical coil. A magnetomotility experiment was conducted on the four different samples to identify the effects of their weight and geometry on their movement. For example, the effect of helix length was evaluated by comparing sample D5-L20 with samples D5-L25 and D5-L35. The helical pitch of D5-L25 was compared with that of D5-L35. In the case of samples with the same pitch or diameter, a comparison can be made according to different weight or length conditions (i.e., D5-L20 and D5-L35).

### 2.2. Method: Rolling Actuation on Flat Surfaces

**Figure 2:**
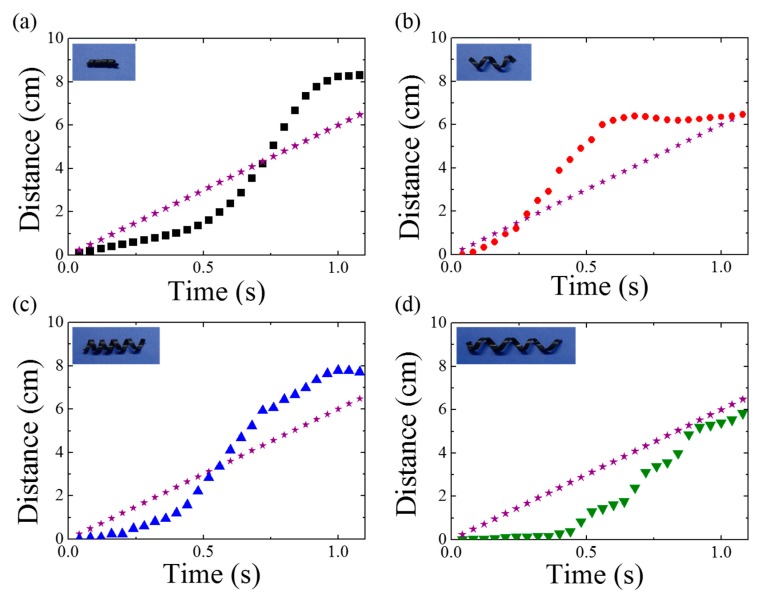
Time-resolved distance information obtained using magnetomotility for helical soft robots of (a) D2-L15, (**b**) D5-L20, (**c**) D5-L25, and (**d**) D5-L35 rolled on a flat surface. The asterisk indicates the travel distance of the magnet underneath the substrate.

First, magnetomotility experiments were conducted on the four samples on a flat surface. Figure 2 presents the time-resolved monitoring of the moving-distance information. The asterisk indicates the moving distance of a magnet underneath the substrate. In the initial stage, the static friction was larger than the magnetic momentum to roll. As the magnet moves along the X-axis, the magnetic momentum of the helix increases along the X-axis, and the rolling motility of the helix is initiated. In the early stage, the stillness inertia is large and the helix rolls slowly, which is followed by a rapid increase in the moving distance. At this stage, the location of the magnet is ahead of the helix. Then, the distance between the magnet and the helix decreases, resulting in a reduction in the magnetic momentum of the helix along the X-axis. As the helix location is finally ahead of the magnet owing to rotational inertia, the magnetic attraction pulls the helix. Therefore, the rolling resistance becomes dominant again, as is evident from the distance plateau of the helix as a function of time. The rolling resistance in the case of D2-L15 was larger than that in the case of D5-L20, because the helix diameter was smaller while the weights of both the samples were the same (i.e., 30 mg). For the same weight, the rolling resistance, F_r_, decreases with increase in diameter according to the following equation:

F_r_ = f_r_ N/R, (1)

where N is the normal force, f_r_ is the rolling resistance coefficient, and R is the cylinder radius. Therefore, D2-L15 requires a larger magnetic momentum and takes a longer time to initiate rapid rolling. Once the D2-L15 helix rolls, the moving distance is greater than that in the case of D5-L20, which has been attributed to the more robust shape retention of the helix owing to the smaller pitch and diameter of D2-L15. 

### 2.3. Method: Magnetomotility Experiments on a Gastric Cancer Model

**Figure 3:**
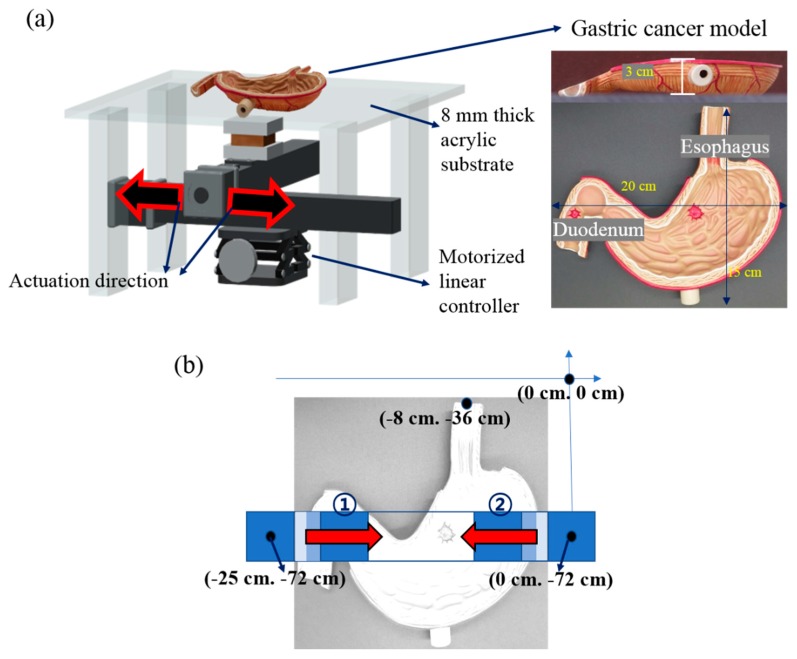
(**a**) Schematic of the experimental setting of the gastric cancer model; (**b**) Structural information of the stomach using the cancer model. Coordinate information for the experiment starts from the esophagus (①) and duodenum (②).

The magnetomotility experiment of the helical soft robots was conducted by linearly moving a permanent magnet (NdFeB, Kingkong Magnet, N35 grade, the magnetic-flux density in the middle of the top surface is 0.2 T) using a linear translational controller (Motorized stage, Sciencetown^TM^) (Figure 3a). The motorized moving stage was placed on an optical table to remove the artifacts originating from the tilt angle of the stage. Considering the potential ethical issue, we used the artificial gastric cancer model (GPI Anatomicals, Lake Bluff, IL, USA) before conducting an animal or human study; the model was placed on an 8-mm-thick acrylic substrate. The magnetomotility of the four different helical soft robots were investigated on the stomach cancer model. As shown in Figure 3b, the directionality of the magnetomotility tests was examined 10 times for each sample type from two different origins—the esophagus and duodenum—in the gastric cancer model. Two different magnet velocities—10 mm/s and 60 mm/s—were employed in the range of 25 cm of the magnet displacement to investigate the magnetomotility of the soft robots according to the two velocities.

**Figure 4:**
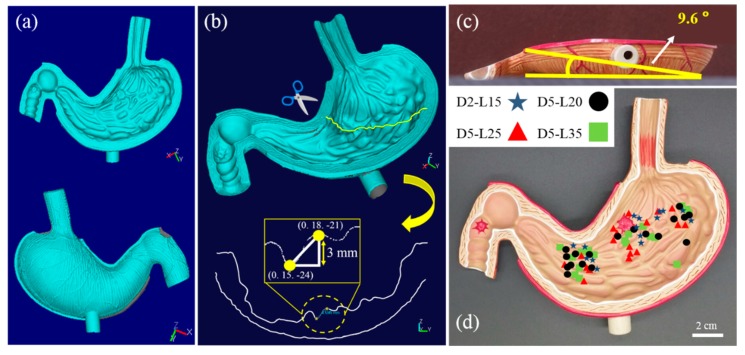
(**a**) Three-dimensional (3D) scanned images from the top and bottom of the gastric cancer model; (**b**) Sectioning and measurement of 3D location information for calculating wrinkle height in the gastric cancer model; (**c**) Angle of the slope of overall gastric cancer model obtained using the coordinate information; (**d**) Cessation location of magnetic actuation indicated for helical soft robots.

Three-dimensional (3D) positional information of the gastric cancer model was measured using a 3D scanner (Laser Design System Inc., Surveyor DS series) with a spatial resolution of 0.020 mm for each voxel and a repeatability of 0.006 mm, as detailed in Figure 4a. The 3D coordinate information of the gastric cancer model was recorded by combining the information from the top–down and bottom–up measurements (Figure 4b). The difference in the z-axis coordinate information of the gastric cancer model provides thickness information of the wrinkles at certain x and y positions. The thickness information of the wrinkles at each coordinate was used to precisely estimate the distance between the soft robot and the magnet; it was also used to calculate the magnetic-flux density applied to the soft robot. In addition to the microscopic wrinkle information, the macroscopic slope of the gastric cancer model was measured to be 9.6°, as shown in Figure 4c. Figure 4d shows the locations where the helical soft robots ceased their magnetomotility in each experiment.

## 3. Results

### 3.1. Rolling Actuation on Flat Surfaces

The digital images of the four different helical soft robots are presented in Figure 1b. The code names and geometric information of the samples (weight, helical pitch, helix diameter, and helix length) are summarized in Table 1. The helical pitch and helix diameter of D2-L15 are 3 mm and 2 mm, respectively, which are the smallest among those of all the samples.

By comparing D5-L20 and D5-L25, the effects of weight and helical pitch on the magnetomotility can be investigated. An increase in weight caused an increase in the effect of inertia at the initial stage, resulting in a delay to reach the critical rapid rolling. However, D5-L25 completed a longer distance after rolling because of the smaller helical pitch. From Figure 2d, a significantly different magnetomotility value was observed. The distinctly different intermittent movement of D5-L35 has been attributed to the local deformation [27] originating from the large length and helical pitch.

### 3.2. Magnetomotility Experiments on a Gastric Cancer Model

**Figure 5:**
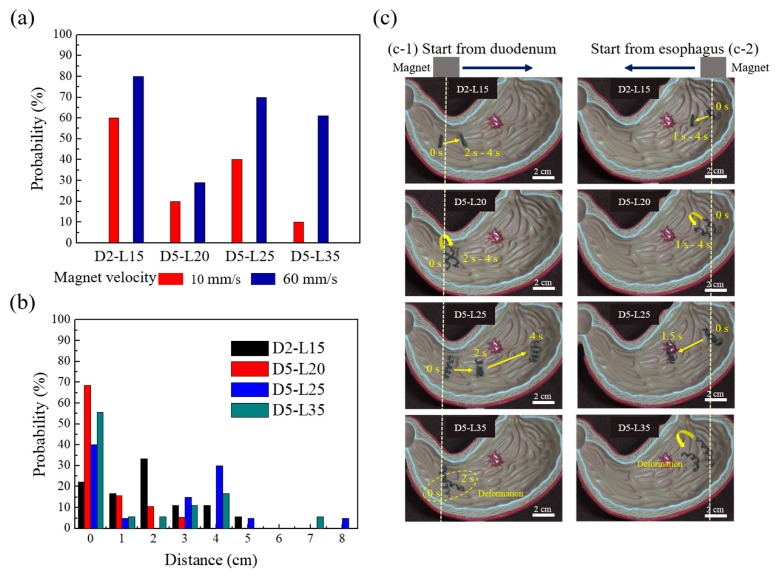
(**a**) Probability of successful magnetic actuation of helical soft robots by exceeding static friction at magnetic velocities of 10 mm/s and 60 mm/s; (**b**) Histogram of traveled distance using magnetic actuation for the four samples; (**c**) Representative images of magnetomotility for the four different samples for esophagus departure (**1**) and for duodenum departure (**2**) cases.

When the experiments were conducted at magnetic speeds of 10 mm/s and 60 mm/s, the samples were significantly less likely to move at a magnetic speed of 10 mm/s, as can be inferred from Figure 5a. Therefore, this study focused on experiments with a speed of 60 mm/s, which has more consistent results and is statistically more robust. Note that 60 mm/s was the mechanical limitation of the linear translational stage used in this study. The time-resolved movement was monitored, and the moving distances were calculated from the x and y positions. When the moving distance was analyzed for the four different helical soft robots, a certain pattern was observed in each experiment as follows: the moving distance of D2-L15 was longer than that of D5-L20 but shorter than that of D5-L25 (Figure 5b,c). Owing to the small helix diameter of D2-L15, all the wrinkles in the stomach acted as obstacles, hampering the forward movement. Since wrinkles with heights larger than the diameter of D2-L15 could not be overcome by the sample, it was difficult for the sample to reach the target point through the given magnetic driving force. D5-L20 showed the least probability for successful movement and the smallest actuating distance (Figure 5b). Despite the higher diameter, the helical pitch was so large for a short coil that local deformation of the helix occurred because of wrinkles, preventing the rolling locomotion. In the case of D5-L25, the farthest distance by magnetomotility was observed, and no noticeable local deformation occurred. As shown in Figure 5c-1, the sample fell in the deep wrinkle at 2 s; however, D5-L25 climbed the wrinkle and continued the rolling locomotion. D5-L35 has the largest length and pitch among the four samples (Table 1). The large helix length resulted in clear local deformation of the helix and inefficient locomotion on the wrinkles. When a locally deformed part has deep wrinkles, the locomotion of the entire helical coil is hindered. Therefore, the probability of failure increases. In addition, the locomotion direction of the deformed part is different from the initial coil, resulting in a competition between two force vectors. Overall, D5-L25 showed the most effective actuation among the four samples, considering the probability for successful actuation and the moving distance (Figure 5b). D2-L15 and D5-L35 achieved a limited locomotion distance, and D5-L20 showed low probability for successful locomotion owing to the local deformation of the helix. 

**Figure 6:**
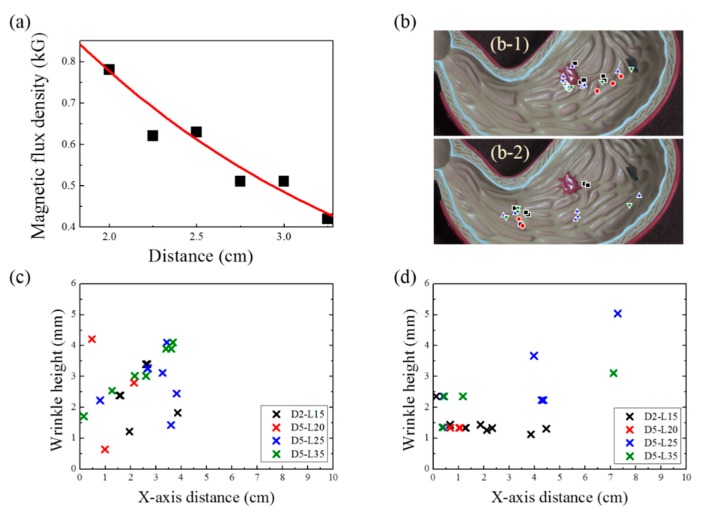
(**a**) Variation in magnetic-flux density with respect to the distance between the magnet and the samples; (**b**) Cessation location of magnetic actuation indicated by the X symbol for esophagus departure (1) and duodenum departure (2) cases; (**c**) Wrinkle height and X-axis distance where the soft-robot magnetic actuation ceased in the case of esophagus departure; (**d**) Wrinkle height and X-axis distance where the soft-robot magnetic actuation ceased in the case of duodenum departure.

To find the range of magnetic-flux densities according to the distance between the sample and the magnet, the exponential decay equation was fitted to the experimental data, as shown in Figure 6a. The equation for the fitting is as follows:

Y = 2.03 × exp (−X/1.92) + 0.06.(2)

In this study, the magnetic-flux densities ranged from 600 to 602 G, which is not deterministic for magnetomotility. Hence, we focused on the geometry of the helix and the height of wrinkles in the 3D gastric model. Figure 6b illustrates the locations of the samples when their magnetic actuations ceased, denoted by X symbols, after the experiments were completed. Figure 6b-1 shows the final positions of the samples, starting from the esophagus, whereas Figure 6b-2 shows the final positions of the samples, starting at the duodenum. The wrinkle height data and the X-axis distance are summarized in Figure 6c and d for Figure 6b-1 and Figure 6b-2, respectively. As previously presented in Figure 4c, the gastric cancer model has an overall slope of 9.6°. This angle generates the inclination and angle for esophagus departure, which results in the consumption of magnetic energy for potential energy. Hence, the helical soft robot cannot travel further than 4 cm along the X-axis. Conversely, the declination angle induces easier rolling for the duodenum departure case; a larger X-axis distance is covered, and higher wrinkles are overcome, which is in conjunction with kinetic inertia effects.

### 3.3. Influence of Helix Geometry

**Figure 7:**
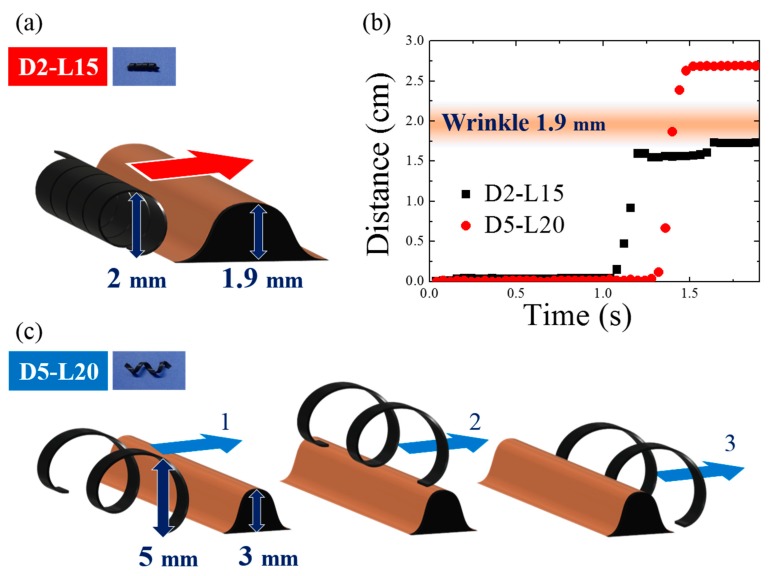
(**a**) Representative scheme for magnetomotility of D2-L15 at 1.9-mm wrinkle height; (**b**) Time-resolved magnetomotility of helical soft robots for D2-L15 and D5-L20 at 1.9-mm wrinkle height. D2-L15 stopped at the wrinkle with a height of 1.9 mm; (**c**) Representative scheme for magnetomotility of D5-L20 overcoming wrinkles with a height of 1.9 mm.

**Figure 8:**
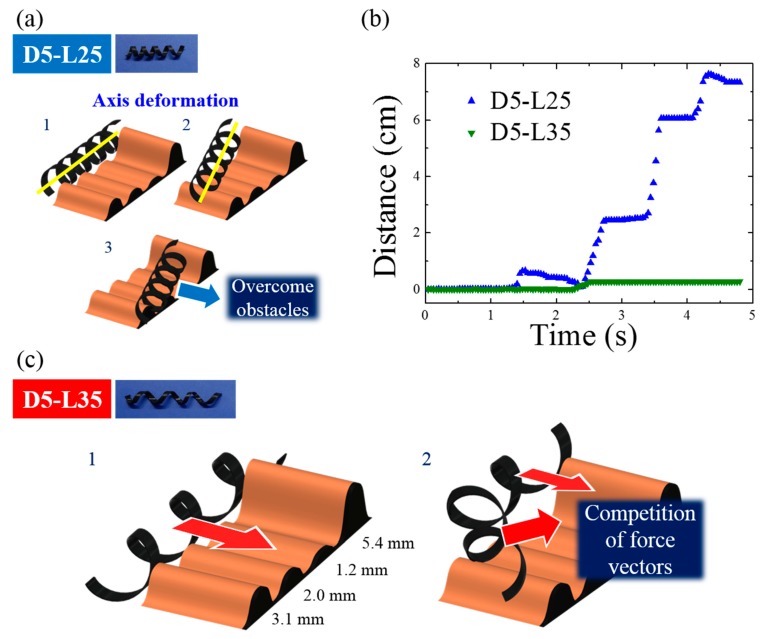
(**a**) Representative scheme for magnetomotility of D5-L25 at four wrinkles with different heights; (**b**) Time-resolved magnetomotility of helical soft robots for D5-L25 and D5-L35 at four different wrinkles; (**c**) Representative scheme for magnetomotility of D5-L35 at four different wrinkles.

Based on the magnetomotility experiments on the gastric model, the representative causes for the cessation of magnetomotility were illustrated and analyzed further, as shown in Figure 7 and Figure 8. Figure 7 compares the magnetic responsivities of D2-L15 and D5-L20. The main difference between the two samples is their diameters. When the helical diameter exceeded the height of a wrinkle, the helix climbed the wrinkle as in the case of D5-L20. In contrast, when the wrinkle height was comparable to the diameter of the helix, the D2-L15 helix was physically blocked by the 1.9-mm obstacles. 

The most important and frequent example of failure is illustrated in Figure 8. During the magnetic locomotion over the wrinkles, D5-L25 retained its helical geometry without noticeable deformation. Instead of local deformation, D5-L25 shifted its axis to circumvent the wrinkles, as shown in Figure 8a. However, D5-L35 has a larger helical pitch and aspect ratio. Shape deformation occurred in D5-L35 because the mechanical local deformation of D5-L35 is easier. This local deformation generates shifts in the center of mass and a collision of the magnetic momentum vectors. As a result, magnetomotility ceased in the case of D5-L35. This example clearly demonstrates the importance of the helical pitch and the aspect ratio of the helical soft robots because both D5-L25 and D5-L35 have the same rolling resistances without local deformations.

## 4. Discussion

This experimental feasibility study examined the fabrication and behavior of a soft-robot model that can be moved to a targeted lesion by the application of an external magnetic field in the stomach. In addition, the suitable structural characteristics of the model in terms of its shape could be identified by assessing its motion to allow it to overcome wrinkles in the stomach. Uniquely, in this study, a helical soft robot that moved even with a small magnetic-flux density in the gastric cancer model was implemented, and a solution for its shape was presented. 

This study examined the appropriate characteristics of a magnetically active helical soft robot in a gastric cancer model. For the soft robot to move freely in the human body, its geometrical parameters, such as its diameter, length, and pitch, are important factors. In addition, natural obstacles inside the human body, such as the mucosal height, must also be considered. In the present study, the size of the mucosal barrier in the stomach at the position of the soft robot was important. For example, of the four samples, the tightly coiled D2-L15 sample had the smallest diameter and length, and one can expect that it can navigate well through a narrow space without local deformation of the helix. In contrast, tight coiling induced a small helix diameter, meaning that the height of the stomach wrinkles that this sample can overcome may be minimal. Indeed, the D2-L15 sample failed to reach the target point by overcoming the wrinkles within the stomach model, because the height of the wrinkle was larger than the diameter of the sample. This indicates that to actuate the soft robot toward the target point easily by overcoming stomach wrinkles, both the helix diameter and stomach wrinkle height must be considered. Otherwise, a different magnetomotility mechanism must be employed. Therefore, the use of in vivo robots with larger helical diameters is more advantageous to overcome the wrinkles of the gastric fold.

Moreover, the helical pitch of the soft robot must be considered in order for soft robots to reach the target lesion successfully. As the value of the helical pitch decreases, the deformation of the helix is suppressed. In the present study, the D5-L25 model exhibited the most efficient magnetic actuation to reach to the target lesion, thereby suggesting that the helix is an efficient shape for movement of soft robots without significant local deformation of the helix. The D5-L25 model has a helical pitch that is comparable to the helix diameter and one-fifth of the length. Overall, by considering the diameter, length, and pitch information, D5-L25 was found to have the optimal geometric specifications in this study. This result can be seen in a Table 2, which is formed by averaging the X-axis distances in Figure 6. These findings suggest that the geometry of the helical soft robot must be designed appropriately by considering the morphological parameters in order for the robot to reach the target lesion.

There were some limitations of this study. First, the biocompatibility of the samples was not assessed. Thus, a biocompatibility study must be conducted as a first step to determine the practical use of the samples. Nevertheless, the present study is meaningful in that it presents the directionality of soft-robot models using a helical shape that is helpful considering the human biology. In addition, the structural characteristics of soft robots applicable to the diagnosis or treatment of certain pathological lesions in the stomach were identified. Second, the autonomic mobility of wrinkles in the stomach was not assessed, even though it is one of the parameters that should be considered when a soft robot moves in the stomach. However, it was impossible to reproduce it using an experimental stomach model; considering the ethical issues, it is necessary to first verify its suitability for application in an animal model before it is directly applied in a human body. Third, the experimental setup in this study was not fully consistent with the actual internal environment of the stomach, wherein there are some rigid and some are moist. Nevertheless, it was demonstrated that soft robots can surpass the height of the wrinkles in a dried environment.

## 5. Conclusions

We identified that the D5-L25 sample was the most suitable model for a helical soft robot that could be moved to a target lesion by magnetic actuation in the stomach. Moreover, this study found that not only the diameter or length of the helical soft robot but also its helical pitch are important factors in the successful arrival of the robot at the target lesion. These findings suggest that helical soft robots can be used to diagnose and treat patients with gastric cancer or gastric ulcers in the future. Nevertheless, the findings of this feasibility study must be validated through real animal models and human studies.

## Figures and Tables

**Table 1:** Weight and dimension information of helical soft robots.

	D2-L15	D5-L20	D5-L25	D5-L35
Weight (mg)	30	30	40	40
Pitch (mm)	3	10	5	10
Diameter (mm)	2	5	5	5
Length (mm)	15	20	25	35

**Table 2:** Summary of Figure 6.

	D2-L15	D5-L20	D5-L25	D5-L35
Average of X-axis distance (cm)	2.1	1.1	3.2	2.4
